# Evaluating the Impact of hydration on electrodermal activity: A Comparison between AC bioimpedance and DC skin potential metrics

**DOI:** 10.2478/joeb-2026-0008

**Published:** 2026-07-23

**Authors:** Ardawan A. Youssif, Dindar S. Bari, Haval Y. Yacoob Aldosky

**Affiliations:** Department of Physics, College of Science, University of Zakho, Zakho, Kurdistan region, Iraq; Directorate of Scientific Research Centers, University of Zakho, Zakho, Kurdistan region, Iraq; Department of Physics, College of Science, University of Duhok, Duhok, Kurdistan region, Iraq

**Keywords:** AC and DC measurements, hydration, skin conductance, skin potential, skin susceptance

## Abstract

This study evaluates the comparative sensitivity and stability of tonic and phasic electrodermal activity (EDA) parameters, specifically skin conductance (SC), susceptance (SS), and potential (SP), following a localized moisturizer intervention. Utilizing a single-group pretest-posttest design (N = 55), the research demonstrates that AC bioimpedance-based metrics (SC and SS) exhibit distinct response dynamics compared to conventional DC-based measures. Tonic-level analysis revealed that SCL and SSL produced nearly identical relative percentage increases (52.6%) compared to SPL (34.46%). While SCL provided the largest absolute change, SSL emerged as the most statistically stable indicator across the cohort, characterized by a highly uniform signal-to-noise ratio (d = 1.23). Phasic analysis revealed that Skin Susceptance Responses (ΔSSRs) demonstrated a highly pronounced downward shift following the intervention, characterized by a −70.2% change from baseline. This negative shift was significantly divergent from the potential response (p < 0.001, d = −0.55), highlighting a distinct directional sensitivity for ΔSSRs under these experimental conditions. These findings suggest that AC-based EDA parameters are highly responsive to the resistive and capacitive shifts within the stratum corneum interface. The results suggest that for applications evaluating localized skin surface changes, a multimodal approach incorporating AC bioimpedance-based metrics may provide a highly responsive framework for detecting acute alterations at the electrode-skin interface compared with traditional potential-based measurements.

## Introduction

The electrical characteristics of the skin have been demonstrated to be strongly influenced by skin hydration [1]. Understanding these changes is essential for improving the interpretation of electrodermal activity (EDA) in physiological and clinical contexts, as skin hydration directly impacts both autonomic nervous system monitoring and skin barrier assessment.

EDA is mediated by sweat gland activity and is commonly used in physiological assessments due to its strong relationship with sympathetic nervous system activity. By creating ionic current pathways through the highly resistive stratum corneum, sweating causes sudden changes in skin conductance (SC) [2]. The EDA consists of two major parameters: tonic (level) and phasic (response), each with various time scales and relationships with stimuli [3]. The tonic parameter—frequently measured as skin conductance levels (SCLs) or skin potential levels (SPLs)—reflects gradual fluctuations over longer timescales (tens of seconds to minutes) and represents general arousal or physiological condition [4]. The phasic parameter consists of brief increases in conductance or potential—called skin conductance responses (SCRs) or skin potential responses (SPRs)—which occur a few seconds after stimuli and reflect temporary sympathetic sudomotor activation [4]. The overall EDA activity level increases when human skin is hydrated because water is a good electrical conductor [5].

Depending on the recording method, EDA parameters are recorded using direct-current (DC) or alternating-current (AC) measurements. Endosomatic measurements are almost always associated with DC recordings because they measure natural voltage differences generated in the skin (SPLs and SPRs) with no current injected [6]. In DC recordings, a constant potential difference is measured across skin electrodes, reflecting changes in skin potential (SP) generated by electrochemical processes at the skin–electrode interface and within the sweat ducts [6]. While commonly used to monitor sympathetic sudomotor activity, DC measurements only quantify skin potential [2, 7]. Because they cannot capture changes in the skin's capacitive properties, DC recordings alone are limited in how deeply they can characterize the structural and dielectric variations caused by hydration.

In contrast, exosomatic AC measurements apply a small alternating current across the skin, allowing the skin's electrical response to be separated into resistive and capacitive components [8]. The resistive parameter corresponds to SC (measured via SCLs and SCRs), while the capacitive component is represented by skin susceptance (SS, measured via SSLs and SSRs), which reflects the dielectric properties of the stratum corneum [9]. Because the skin behaves electrically as a distributed resistor–capacitor (RC) system, AC measurements detect impedance and capacitive properties [8]. Because skin hydration affects both ionic conduction and dielectric capacitance of the stratum corneum, AC bioimpedance—by quantifying both conductance and susceptance—offers a richer, more detailed characterization of hydration status and barrier properties than DC measurements alone [2, 7]. Consequently, AC and DC electrodermal measurements provide complementary information regarding skin pick-up parameters related to sweat gland activity, skin hydration, and autonomic nervous system function.

This work focuses on determining the impact of hydration on EDA's DC and AC parameters. The aim is to directly compare AC bioimpedance-based EDA (SC and SS) with DC-based measurements (SP) in their ability to detect hydration-induced changes. Specifically, we evaluated which modality exhibits greater relative sensitivity across both tonic and phasic parameters.

While skin hydration is known to influence both AC [10, 11] and DC [12] electrodermal measures, a direct experimental comparison between AC and DC techniques under well-regulated, controlled hydration conditions is virtually non-existent. Specifically, it remains unknown whether AC bioimpedance provides enhanced sensitivity or truly complementary information compared to DC measurements during real-time hydration changes.

This work focuses on determining the impact of hydration on EDA's DC and AC parameters. The aim is to directly compare AC bioimpedance-based EDA (SC and SS) with DC-based measurements (SP) in their ability to detect hydration-induced changes. Specifically, we evaluated which modality exhibits greater relative sensitivity across both tonic and phasic parameters.

## Materials and methods

### Measurements

The three-electrode system, which enables simultaneous measurement of SC, SP, and SS, as described in [13, 14] was used. This setup includes a front-end electronic box connected to a data acquisition (DAQ) card that interfaces with a LabVIEW-based PC laptop. The system comprised a measuring electrode, a reference electrode, and a current-sink electrode. The measuring electrode and the current-sink electrode facilitated AC measurements for SC and SS in units of μS, while the DC parameter (SP) was measured in units of mV between the measuring and reference electrodes. A 200-mV sine wave was generated by the DAQ Card and fed to the Howland (current source) circuit, which in turn delivered a 20 Hz alternating current of about 20 μA to the skin. The DAQ card received the analog signals (10 samples per second) back from the skin via the front-end electronic box and converted them to digital form. The digitized signals were then processed by differentiation in LabVIEW and separated into a DC component for SP and an AC component for SC and SS from the skin admittance signal by phase-sensitive rectification.

The employed electrodes were Kendall Kittycat 1050NPSM Ag/AgCl solid gel ECG neonatal electrodes with an active electrode area of 5.05 cm^2^. The three electrodes were placed on one arm of the participants. The measuring electrode was placed on the hypothenar site of the palm, the reference electrode was attached on the apex of the elbow, and finally the current-sink electrode was placed on the underarm between the measuring and reference electrodes.

### Study protocol

For the current study, 55 apparently healthy subjects, ranging in age from 19 to 43 years, with a mean age of 27 years, were recruited. Before participating in the study, they willingly completed a written informed consent form. During both periods, each test subject was seated in a comfortable chair in a serene setting with a typical temperature of 22 to 23 °C. Additionally, they were told to remain calm and not move. Before the test, the volunteers were also asked not to wash their hands or do any physical exercise. Subjects were exposed to a standardized digital auditory stimulus (an acoustic handclap through the computer speakers for one second) to induce mental stress and elicit SCRs, SPRs, and SSRs. Electrode stabilization was permitted for five minutes before the commencement of measurements in both sessions. In the first session, EDA measurements were taken from subjects' hands in a normal skin state without moisturizer. In the second session, local skin hydration was experimentally induced on the participants' hands via the application of a moisturizer. 0.2 grams of a 0.5% KCl with a 2% agar formulation was applied to the hand, and 90 seconds were allowed for equilibration. The moisturizer was applied to the skin areas where the measuring and the current-sink electrodes were attached; specifically, it was applied on the hypothenar site of the palm (measuring electrode site), and on the underarm (current-sink electrode site). It is important to note that this local intervention concurrently introduces moisture, exogenous electrolytes, and changes to the electrode-skin coupling interface. This session was performed with the electrodes placed on the moisturized skin areas, and after five minutes, the EDA signals were recorded.

### Data and statistical analysis

To compute phasic parameters, the onsets and peaks of SCRs, SSRs, and SPRs were first identified, and then the amplitudes of the SCRs, SSRs, and SPRs were calculated from the differences between the onsets and peaks of SCRs, SSRs, and SPRs, respectively. Tonic parameters (SCL, SSL, and SPL), on the other hand, were obtained from the onsets of the responses coming from the sound stimulus.

To evaluate multi-factor relationships, a within-subject repeated-measures design was used, in which each participant underwent both baseline and hydrated recordings. This approach allowed the data analysis to capture individual within-subject changes across multiple interacting factors, specifically comparing measurement types (AC vs. DC), hydration states (pre- vs. post-hydration), and EDA parameters (phasic vs. tonic). To test whether AC bioimpedance provides enhanced or complementary sensitivity to skin hydration compared with DC measurements, the percentage of mean changes in hydration status was used. The percentage change of the mean was calculated by subtracting the mean pre-hydration value from the mean post-hydration value, dividing the result by the mean pre-hydration value, and multiplying by 100.

Effect sizes for paired comparisons between change variables were estimated using Cohen's d for paired samples (within-subject change scores). Cohen's d was calculated by dividing the mean difference between variables by the standard deviation of the paired differences. Normality of the within-subject change scores (Δ = post − pre) was assessed using the Shapiro–Wilk test. As the assumption of normality was not satisfied, paired comparisons were performed using the Wilcoxon signed-rank test.

Statistical significance was evaluated at *p* < 0.05 (two-tailed), and the 95% confidence intervals (CI) of the differences were calculated for all mean comparisons to provide an estimate of the precision and magnitude of the observed effects. All analyses were performed using IBM SPSS Statistics.

### Informed consent

Informed consent has been obtained from all individuals included in this study.

### Ethical approval

The protocol has complied with all relevant national regulations, institutional policies, and in accordance with the tenets of the Helsinki Declaration and has been approved by the Research Ethical Committee (ethics approval number: FoS.UoZ/8/9/2024) at the University of Zakho.

## Results

### Phasic (response amplitude)

The mean and effect size for the phasic parameters before and after the hydration are presented in Table 1. Following the localized intervention, the metrics demonstrated clear percentage shifts relative to their baseline means. The skin conductance responses (SCRs) increased by 29.69%, representing a change of about 1/3 of baseline with a Cohen's d of 0.37. For the parameters with negative baselines, the skin susceptance response (SSRs) exhibited a 70.23% shift deeper into the negative spectrum (moving from −1.31 to −2.23 μS), corresponding to approximately a 3/4 change relative to the baseline value, and Cohen's d of −0.56, which indicates a medium-to-large decrease following intervention. Conversely, the skin potential responses (SPRs) showed a 23.25% reduction in their negative magnitude (moving from −5.72 to −4.39 mV), tracking a localized depolarization at the interface. To maintain physical clarity, the signs of the percentage changes for SSRs and SPRs directly reflect their directional physiological movement rather than raw algebraic quotients.

**Table 1.** Mean and effect size for the phasic EDA parameter pre- and post-hydration.

EDA parameter	Mean Pre-hydration	Mean post-hydration	% Change ofmean (Δ)	Effect Size-Cohen’s d^†^	Interpretation
SCR	13.64	17.69 *	29.69	0.37	Small change
SSR	−1.31	−2.23 *	−70.2	−0.56	Medium-to-large change
SPR	−5.72	−4.39 *	−23.25	0.33	Small change

* *Wilcoxon signed ranks test P < 0.05 with respect to mean pre-hydration.*

† *The effect sizes are small when (d = 0.2), medium (d = 0.5), and large (d = 0.8).*

**Fig. 1:**
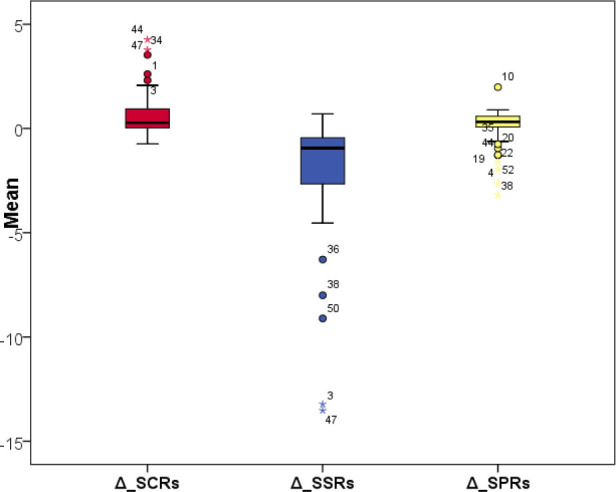
The box plot distribution of the mean change in phasic EDA parameter pre- and post-hydration.

Figure 1 displays the distribution of mean changes across all subjects for each phasic EDA parameter. The distribution of ΔSCRs was situated almost entirely above the zero baseline, demonstrating an increase in skin conductance across the cohort. Conversely, for ΔSSRs, both the box and median were positioned below the zero baseline, showing a negative shift in skin susceptance. The distribution of ΔSPRs clustered closer to the zero line, indicating a lower magnitude of shift. Regarding distribution spread, ΔSCRs and ΔSPRs exhibited narrower interquartile ranges compared to the wider distribution and presence of individual outliers observed in the ΔSSRs data.

As shown in Table 2, the negative medium-to-large effect size for ΔSSRs and ΔSPRs, despite being in the opposite direction, exhibits a clearer statistical separation under this protocol than DC-based skin potential. The ΔSSRs not only differ numerically from the ΔSPRs, but they also represent a statistically superior shift in magnitude (*p* < 0.001). A small effect size for the pair (ΔSCRs – ΔSPRs) indicates a subtle, limited difference between two paired groups or conditions, suggesting that while a result might be slightly different, the change in (ΔSCRs) and (ΔSPRs) is almost the same, and it is not practically important. A Small to medium effect size was observed in the AC-based EDA parameters, particularly for ΔSCRs and ΔSSRs following hydration.

**Table 2.** Comparison between the mean change of phasic EDA parameters and their effect size.

Comparison	p-value	Effect Size-Cohen’s d †	95% Confidence Interval of the Difference		Interpretation

			Lower	Upper	
Δ_SSRs vs Δ_SPRs	0.001	−0.55	− 3.31726	− 1.13365	Medium-to-large change
Δ_SCRs vs Δ_SPRs	0.101	0.23	− 0.55200	6.04254	Small change
Δ_SCRs vs Δ_SSRs	0.003	0.42	1.75261	8.18885	Small-to-medium change

† *The effect sizes are small when (d = 0.2), medium (d = 0.5), and large (d = 0.8)*

### Tonic (level)

The mean and effect size for tonic parameters before and after the hydration are presented in Table 3. SCL increased from a pre-measurement mean of 41.31 to a post-measurement mean of 63.04 with a very large Cohen’s d of 1.13. The mean change percentage was 52.60%, which indicates an increase of around half of the baseline. Similarly, SSL increased from a pre-measurement mean of 8.22 to a post-measurement mean of 12.54. The mean change percentage was 52.55%, which also indicates an increase of around half of the baseline, with the highest effect size of 1.23. In contrast, SPL decreased from −18.31 at pre-measurement to −12.00 at post-measurement. The mean percentage change was −34.46, a decrease in the size of the negative baseline, which is equivalent to a decrease of about 1/3 of the baseline. Generally speaking, the results demonstrate highly significant (*p* < 0.001) increases in SCLs and SSLs from pre- to post-hydration, whereas SPL showed a highly significant (*p* < 0.001) reduction in magnitude following hydration.

**Table 3.** Mean and Effect Size for the tonic EDA parameter pre- and post-hydration.

EDA parameter	Mean Pre-hydration	Mean post-hydration	% Change of mean (Δ)	Effect Size-Cohen’s d†	Interpretation
SCL	41.31	63.04 *	52.60	1.13	Very large change
SSL	8.22	12.54 *	52.55	1.23	Very large change
SPL	−18.31	−12.00 *	−34.46	0.84	Large change

* *Wilcoxon signed ranks test P < 0.05 with respect to mean pre-hydration.*

† *The effect sizes are small when (d = 0.2), medium (d = 0.5), large (d = 0.8), and very large (d > 0.8).*

**Table 4.** Effect Size for the change of tonic EDA parameter.

Comparison	p-value	Effect Size-Cohen’s d †	95% Confidence Interval of the Difference		Interpretation

			Lower	Upper	
Δ_SCL vs Δ_SPL	0.001	+ 0.67	9.16770	21.66897	Medium
Δ_SSL vs Δ_SPL	0.157	− 0.23	−4.33798	0.36575	Small
Δ_SCL vs Δ_SSL	0.001	+ 1.00	12.64763	22.16126	Very large

† *The effect sizes are small when (d = 0.2), medium (d = 0.5), and large (d = 0.8), and very large (d > 0.8).*

**Fig. 2:**
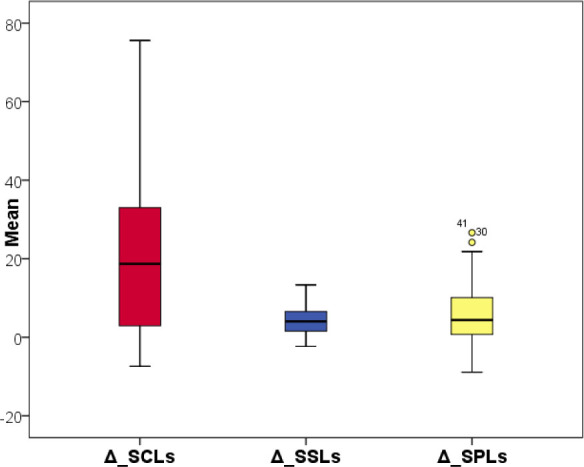
The box plot distribution of the mean change in tonic EDA parameter pre- and post-hydration.

Figure 2 shows the distribution, central tendency, and outliers for all three parameters, ΔSCL, ΔSSL, and ΔSPL. The ΔSCL box is the largest and highest on average. This distribution in the tonic conductance parameter exhibited the widest range of responses and the highest median increase relative to the other parameters. The long upper whisker indicates that a subset of “hyper-responders” experienced very large increases in conductance following hydration. The ΔSSL box, in contrast, is the most compressed. This indicates that the susceptance response was highly consistent across the subject pool with relatively low variability. Even though its absolute magnitude is lower than that of ΔSCL, its narrow distribution suggests it is a very stable and reliable marker of hydration status. Despite having noticeable outliers, the ΔSPL distribution is relatively symmetrical. These data points show particular subjects whose electrical potential response was much higher than the group average, most likely as a result of particular physiological characteristics.

A comparative analysis of the tonic parameters demonstrates that SCL is the most sensitive metric for detecting hydration-induced changes among the levels studied. The statistical comparison reveals a very large effect size (d = 1.00) when contrasting SCL with SSL, and a medium effect size (d = 0.67) when compared to SPL, confirming that SCL exhibits a significantly more pronounced statistical contrast than either counterpart. Conversely, the difference between SSL and SPL was not statistically significant, with a small effect size (d = −0.23), indicating that these two parameters exhibit a relatively comparable sensitivity to the physiological intervention. Consequently, while all parameters reflect hydration shifts, SCL exhibits the highest absolute responsiveness within this tonic-level hierarchy.

## Discussion

Results showed that hydration caused different patterns of changes in the AC and DC parameters of EDA.

### Phasic (Response-Amplitude)

The findings suggest a fundamental difference in how various electrodermal metrics capture the biophysical effects of a localized moisturizer intervention. The disproportionately high reactivity of ΔSSRs compared to traditional metrics supports the hypothesis that AC-based susceptance is uniquely tuned to the dielectric properties of the stratum corneum [15, 16]. While ΔSCRs effectively track the bulk volume of sweat in the ducts [17]. The −70.2% shift in ΔSSRs likely reflects rapid alterations in the capacitive load and ionic density of the skin tissue itself—changes that are captured more readily by susceptance than by the slower, more centralized responses of conductance or potential.

The lower effect size and impact ratio of ΔSPRs (d = 0.33) further imply that the electrical potential is more stable and less susceptible to localized surface fluctuations. Consequently, for research targeting acute skin surface alterations, phasic AC parameters, particularly susceptance-based measurements, demonstrate a heightened statistical sensitivity to local skin surface changes under this protocol compared to traditional DC-based EDA parameters.

A small effect size for ΔSCRs − ΔSPRs means the difference between how SCRs and SPRs change is minor under this stimulus. Given that the activation of the sympathetic nervous system stimulates sweat glands in the skin, which in turn increases electrical conductance, and decreases the SPRs because localized hydration limits the resistance decrement of the sweat gland pathway related to a response [18]. A small effect size for ΔSCRs − ΔSPRs indicates that the stimulus elicits comparable response intensities across these two metrics. Even though ΔSCRs and ΔSSRs are both AC-based electrodermal measurements, they represent different biophysical components of the skin interface, explaining their moderate correlation.

The most significant statistical divergence in this study was observed between ΔSSRs and ΔSPRs (p < 0.001, d = −0.55), indicating a medium-to-large effect size and underscoring a fundamental difference in how these parameters capture the skin's response to the moisturizer interface. This divergence supports the argument that AC-based susceptance is more sensitive to the dielectric changes in the stratum corneum than traditional DC-based potential metrics. While SCRs and SPRs are primarily driven by sudomotor activity and ionic transport [19, 20], the 70.2% highlights a distinct directional sensitivity. The unique capacity of AC-based susceptance allows it to rapidly detect local capacitive fluctuations within the stratum corneum that remain largely undetected by DC-based potential.

The pronounced negative shift observed in ΔSSRs suggests that skin susceptance possesses a distinct directional sensitivity to interface shifts compared to conductance metrics. Because ΔSSRs proved to be highly divergent from both other metrics (p < 0.003 in both cases), it stands out as a highly reactive indicator for rapid interface changes. This sensitivity aligns with previous research suggesting that susceptance measurements at specific AC frequencies can interact with surface layers to reflect immediate structural changes [5]. Furthermore, the wider interquartile range and prominent outliers in the ΔSSRs distribution highlight substantial individual variability in susceptance responses, which may reflect varying baseline skin barrier characteristics or localized physiological sensitivity to hydration within the cohort. In contrast, the tighter interquartile ranges of both ΔSCRs and ΔSPRs indicate a highly uniform sudomotor response across the group. These distinct dynamics suggest that for biophysical applications requiring high-sensitivity monitoring of localized surface shifts, AC-based EDA parameters provide a more responsive framework than DC measurements.

### Tonic (levels)

The results for the EDA tonic level parameters provide a compelling argument for the differential sensitivity of skin electrical properties to the local moisturizer intervention. The most prominent finding is that SCL acts as the primary and most reactive indicator among the tonic levels [4]. The large effect size (d = 1.00) when comparing the pairwise difference of ΔSCLs to ΔSSLs suggests that this statistical contrast is likely driven by the combined effects of sweat duct filling and increased surface moisture, which heavily influence absolute interface impedance [5]. While ΔSCL showed a medium-to-large superiority over ΔSPL (d = 0.67), notably ΔSCL and ΔSSL exhibited nearly identical percentage increases (52.6% vs 52.55%) relative to their own baselines. This indicates that while the absolute magnitude of change is higher in conductance, the relative impact of hydration on the skin’s dielectric properties (i.e., SSL) is equally substantial. The lack of a statistically significant difference between the ΔSSLs and ΔSPLs (*p* = 0.157) suggests that these two parameters possess comparable response intensities. However, the boxplot data showed that ΔSSLs provided the most uniform and consistent signal across the cohort. The high Cohen’s d values across all level parameters (ranging from 0.84 to 1.23) confirm that the localized moisturizer intervention produces a highly standardized shift in tonic EDA, reinforcing the reproducibility of these metrics under this specific protocol.

While ΔSCL and ΔSSL exhibited nearly identical percentage increases relative to their respective baselines, a comparative analysis revealed a statistically significant difference in their response characteristics. The large effect sizes indicate that both ΔSCLs and ΔSSLs demonstrate a substantial magnitude of change in standardized units following intervention. The slightly higher Cohen’s d observed for ΔSSLs (d = 1.23) compared to ΔSCLs (d = 1.13) reflects a stronger signal-to-noise ratio, driven primarily by the lower standard deviation and higher consistency of the susceptance measurements across the study population rather than a greater absolute magnitude of response.

The comparison between ΔSCLs and ΔSPLs shows a highly significant difference with a medium effect size. Although both parameters increased after intervention, the change in conductance demonstrated a statistically larger magnitude of standardized change compared to the change in potential. This suggests that intervention has a more immediate and powerful effect on sweat duct filling and interface coupling than it does on the ionic gradients across the membrane potential. Thus, conductance provides a more pronounced absolute response for hydration than the resting potential [21]. The comparison between ΔSSLs and ΔSPLs did not reach statistical significance and was accompanied by a small effect size. Consequently, a distinct difference in their response intensities could not be confirmed under the present experimental conditions. This suggests they may capture a similar depth of tissue interaction, whereas SCLs capture a more superficial alteration at the interface. Ultimately, the findings show that AC-based measurements offer complementary utility. While SCLs are optimal for identifying the presence of a high-magnitude surface shift due to their large absolute response, SSLs are optimal for precisely quantifying that shift across a wide range of individuals due to their low baseline variability.

### Limitations of the study

While the findings are statistically reliable, the following limitations should be acknowledged to contextualize the results:

1. The current study utilized a single group of 55 subjects. The lack of classification by age, skin thickness, or ethnic background (e.g., Fitzpatrick skin type) limits the generalizability of the findings, as skin dielectric properties and sweat gland density vary across these demographics.
2. A primary limitation of the study is that the KCl moisturizer confounds pure hydration measurements by simultaneously introducing exogenous ions and reducing contact impedance. Future work requires non-electrolytic or sham controls to isolate these interface effects.
3. The lack of an external, non-electrical standard (e.g., corneometry or transepidermal water loss) to independently verify skin state. Therefore, the results strictly demonstrate that localized electrical shifts occur at the electrode-skin interface, rather than reflecting confirmed, generalized physiological hydration changes.
4. The research focused on the immediate shift between pre-test and post-test states. Consequently, the study does not account for the longitudinal decay of the hydration effect or for how these parameters behave during a slow, natural dehydration process over several hours.
5. Additionally, the fixed pre/post design introduces potential confounding variables such as temporal order effects, electrode stabilization, and stimulus habituation. While the auditory stimulus was digitally standardized to eliminate delivery variability, habituation to the repeated cue cannot be entirely ruled out as a factor in the comparative phasic response magnitudes.
6. The high variability observed in the SPL data confirms the fact that resting potential is highly subject-dependent. Future studies would benefit from a larger sample size or a crossover design to further stabilize these signals.

## Conclusion

This study characterizes the varying degrees of statistical sensitivity observed across both tonic and phasic electrodermal activity parameters following a localized moisturizer intervention. The results demonstrate that under this local intervention protocol, AC-based measures (SCL, SSL, and ΔSSR) exhibit a significantly larger standardized magnitude of change compared to traditional DC-based measures (SPL and SPR). These findings suggest that AC parameters may offer a highly responsive framework for evaluating local electrode-skin interface alterations, which may be relevant for optimizing the configuration of electrodermal activity devices during localized skin assessments. However, further research utilizing independent physiological benchmarks is required to determine how precisely these metrics map to deep-tissue physiological states. These dynamics may have practical implications for the optimization of sensor configurations in electrodermal activity devices when accounting for local electrode-skin interface changes.
